# Vulval Lichen Sclerosus Associated With Immune Checkpoint Inhibitor Therapy: A Case Series of 11 Patients

**DOI:** 10.1111/ajd.70058

**Published:** 2026-02-02

**Authors:** Sophie Walter, Francesca Watts, Raquel Ruiz Araujo, Thevaki Wain, Amanda Glanz, Diona Damian, Matteo S. Carlino, Alexander M. Menzies, Georgina V. Long, Linda K. Martin

**Affiliations:** ^1^ School of Clinical Medicine, Faculty of Medicine and Health University of New South Wales Sydney Australia; ^2^ Department of Diagnostic Oncology and Tissue Pathology Royal Prince Alfred Hospital Sydney Australia; ^3^ Faculty of Medicine and Health The University of Sydney Sydney Australia; ^4^ Westmead Hospital Sydney Australia; ^5^ Dermatology Department Royal Prince Alfred Hospital Sydney Australia; ^6^ Sydney Melanoma Diagnostic Centre Sydney Australia; ^7^ Melanoma Institute Australia The University of Sydney Sydney Australia; ^8^ Blacktown Hospital Sydney Australia; ^9^ Mater Hospital Sydney Australia; ^10^ Royal North Shore Hospital Sydney Australia; ^11^ Charles Perkins Centre The University of Sydney Sydney Australia

**Keywords:** adverse effect, dermatology, genital, immune checkpoint inhibitor, lichen sclerosus, melanoma, vulval

## Abstract

There have only been a limited number of case reports that have described vulval lichen sclerosus in patients receiving immune checkpoint inhibitor (ICI) therapy. We describe 11 cases of vulval lichen sclerosus in patients with melanoma treated with ICIs, and in nine of these cases, the lichen sclerosus symptoms began after ICI commencement. This represents the largest reported series to date and highlights the need for clinician awareness of this potential immune‐related adverse effect.

## 1 Introduction

Immune checkpoint inhibitor (ICI) therapy is a form of immunotherapy that is widely used in treating melanoma and other malignancies [1]. ICIs, which include PD‐1 inhibitors (e.g., nivolumab and pembrolizumab), CTLA‐4 inhibitors (e.g., ipilimumab) and LAG‐3 inhibitors (e.g., relatlimab), augment antitumour immunity by inhibiting immune checkpoint pathways that suppress T‐cell activity [1]. While efficacious in cancer treatment, ICIs are frequently associated with a broad spectrum of immune‐related adverse events (irAEs), including dermatological toxicities [1].

Among the wide range of potential cutaneous adverse effects caused by ICIs, there have been a limited number of reports of vulval lichen sclerosus, a chronic inflammatory dermatosis with autoimmune features [2, 3, 4, 5, 6]. There have also been reports of genital lichen sclerosus in males on ICIs and descriptions of extragenital lichen sclerosus in men and women on these treatments [3]. Here we report cases of vulval lichen sclerosus in patients who received ICIs for melanoma, including women whose lichen sclerosus began after ICI commencement and women with pre‐existing disease.

## 2 Methods

We retrospectively reviewed the Melanoma Research Database of Melanoma Institute Australia (MIA) to identify patients with lichen sclerosus at any time from October 2009 to June 2024. Additional cases of lichen sclerosus during this period were identified via dermatologists and medical oncologists at or collaborating with MIA. Only female patients were included. Other inclusion criteria were clinically or histologically confirmed vulval lichen sclerosus and treatment with at least one ICI. Medical records were reviewed to ascertain demographic characteristics, including age at lichen sclerosus diagnosis, melanoma primary site, stage and ECOG performance status, type and duration of ICI(s), characteristics of vulval lichen sclerosus (latency to lichen sclerosus onset, treatment, response to treatment), toxicities associated with ICI(s) and oncological response to ICI(s). Identified patients were also invited by their treating clinician to complete a structured questionnaire focusing on lichen sclerosus, including clinical features, diagnostic method, clinician making diagnosis, response to treatment and disease‐specific quality of life index (Vulval Quality of Life Index [VQLI]) (Supporting Information S1). For any patient who did not complete the questionnaire, specific information covered by the questionnaire was sought from the medical record. For biopsy‐confirmed cases, histopathology was reviewed and scored by an anatomical pathologist (FW) using a checklist of classical vulval lichen sclerosus features and features that may be atypical (Supporting Information S2). The study received ethics approval.

## 3 Results

Among 1056 female patients receiving ICIs for melanoma between 2009 and 2024, 11 cases of vulval lichen sclerosus were identified (Supporting Information S3). One of the patients had been described in a case report [3]. Characteristics of the 11 patients are summarised in Tables 1 and 2.

**TABLE 1:** Melanoma clinical and treatment characteristics of 11 female patients with vulval lichen sclerosus associated with immune checkpoint inhibitor therapy.

Characteristics	*N* = 11
Age (years) at lichen sclerosus diagnosis, median (range)	58 (47–81)
Melanoma stage, *n* (%)	
Stage III	4 (36)
Stage IV	7 (64)
ECOG Performance Status at time of melanoma diagnosis, *n* (%)	
0	9 (82)
1	2 (18)
2	0 (0)
3	0 (0)
4	0 (0)
Immune checkpoint inhibitor therapy, *n* (%)	
Anti‐PD‐1 monotherapy	6 (55)
Pembrolizumab	3 (27)
Nivolumab	3 (27)
Combination	5 (45)
Nivolumab and ipilimumab	2 (18)
Pembrolizumab and quavonlimab	1 (9)
Nivolumab and BMS‐986249 (anti‐CTLA‐4 probody)	1 (9)
Nivolumab and relatlimab	1 (9)
Duration of immune checkpoint inhibitor therapy (weeks), median (range)	49 (0.14–141)
Concomitant autoimmune side effect, *n* (%)	11 (100)
Thyroid dysfunction	4 (36)
Hypophysitis	3 (27)
Vitiligo	3 (27)
Colitis	2 (18)
Pneumonitis	2 (18)
Pancreatitis	1 (9)
Uveitis	1 (9)
RECIST cancer response to immunotherapy, *n* (%)	
Complete response	4 (36)
Partial response	3 (27)
Progression	4 (36)

Abbreviations: ECOG, Eastern Cooperative Oncology Group; RECIST, Response Evaluation Criteria in Solid Tumours.

**TABLE 2:** Clinical features, method of diagnosis and quality of life impact of vulval lichen sclerosus in 11 patients treated with immune checkpoint inhibitors.

Vulval lichen sclerosus characteristics	*N* = 11
Onset of lichen sclerosus relative to immunotherapy, *n* (%)	
Pre‐immunotherapy	2 (18)
Post‐immunotherapy	9 (82)
Time since histological diagnosis of vulval lichen sclerosus to initiation of immunotherapy in the two pre‐existing cases (months)	26, 106
Time to onset of lichen sclerosus after immunotherapy (months), median (range)	15 (1–39)^a^
Method of diagnosis of vulval lichen sclerosus, *n* (%)	
Clinical	5 (45)
Clinical + histopathological	6 (55)
Initial diagnosing clinician, *n* (%)	
General practitioner	4 (36)^b^
Dermatologist	4 (36)
Gynaecologist	2 (18)
Sexual health physician	1 (9)
Median Vulval Quality of Life Index (VQLI) score, median (range)	10 (0–32)^c^
Symptoms at diagnosis, *n* (%)	
Itch	11 (100)
Soreness	7 (64)
Bruising more easily than before	2 (18)
Burning sensation	4 (36)
Fragile skin	6 (55)
Difficulty with urination	1 (9)
Other	5 (45)
Dyspareunia	1 (9)
Perianal itch	1 (9)
Frequent urinary tract infections	1 (9)
White discolouration	2 (18)

^a^ Calculated for the nine patients with post‐immune checkpoint inhibitor vulval lichen sclerosus.

^b^ Clinical diagnoses of lichen sclerosis initially made by a general practitioner were corroborated clinically by a dermatologist, gynaecologist or sexual health physician.

^c^ For 10 patients, VQLI score completed as part of questionnaire. For the one patient who did not complete the questionnaire, the VQLI score was obtained from the medical record from most recent clinical follow‐up.

Median age at diagnosis of lichen sclerosus was 58 years (range: 47–81 years). Two of the 11 patients had a diagnosis of lichen sclerosus prior to commencing ICI therapy. One of the two patients with pre‐existing vulval lichen sclerosus had worsening of symptoms after initiation of immunotherapy; the second patient had no change in symptoms.

Immunotherapy included anti‐PD‐1 monotherapy, combination anti‐PD‐1 and anti‐CTCL‐4 therapy, or combination anti‐PD1 and anti‐LAG‐3. Four patients had a complete melanoma response to ICI therapy.

All patients experienced at least one other concomitant irAE, which included thyroid dysfunction, vitiligo, hypophysitis, colitis and pneumonitis. Seven of the patients were described in the research database as having another cutaneous toxicity, including pruritus or skin rash not otherwise specified. Further morphological classification of the dermatological eruption was unavailable. One patient had a history of coeliac disease; no other patients reported pre‐existing autoimmune disease.

The most common clinical feature of vulval lichen sclerosus at diagnosis was pruritus.

Diagnosis of vulval lichen sclerosus was confirmed histologically in six patients, including the two pre‐ICI cases and four post‐ICI cases. One of the six patients had a punch biopsy collected at two different time points. All demonstrated classical histological features of vulval lichen sclerosus (Table 3) (Supporting Information S4). One of the 11 patients was diagnosed with a melanocytic tumour adjacent to vulval lichen sclerosus, which was determined to be a new primary melanoma.

**TABLE 3:** Histopathological features of vulval lichen sclerosus in six patients receiving immune checkpoint inhibitor therapy.

Case	1	2	3	4	5*	5*	6
Time of onset of lichen sclerosus relative to immune checkpoint inhibitor therapy commencement	**Pre**	**Pre**	**Post**	**Post**	**Post**	**Post**	**Post**
Lichenoid inflammation	Mild	Mild	Mild to focally moderate	Moderate	Moderate	Mild	Moderate
Vacuolar interface change	Absent	Absent	Absent	Mild	Mild	Absent	Absent
Hyaline fibrosis	Moderate	Mild	Absent	Mild	Moderate	Absent	Moderate
Dermal oedema	Absent	Marked	Moderate	Moderate	Mild	Marked	Marked
Band like inflammation deep to sclerosis	Absent	Present	Absent	Mild	Absent	NA	Moderate to marked
Epidermal atypia	Absent	Mild	Absent	Absent	Absent	Absent	Absent

* Biopsies were collected from case 5 at two different time points, separated by 11 months.

The vulval lichen sclerosus of all patients was treated with topical corticosteroids (*N* = 11). The most potent corticosteroid for each patient was clobetasol propionate 0.05% (*N* = 5), betamethasone dipropionate 0.05% (*N* = 5) and methylprednisolone aceponate 0.1% (*N* = 1). Most patients (*N* = 9) had improvement in symptoms with treatment; one had complete resolution, and one worsened despite treatment.

The median VQLI score, using the most recently available score for each patient, was 10 (range 0–32) out of a possible score of 45.

## 4 Discussion

This case series highlights vulval lichen sclerosus as a potentially under‐recognised cutaneous irAE of ICI therapy. Although idiopathic vulval lichen sclerosus is not uncommon in postmenopausal women, the timing of onset after ICI initiation in previously unaffected patients suggests immune activation as a possible precipitating factor.

Lichen sclerosus is a chronic, lymphocyte‐mediated inflammatory dermatosis with a predilection for anogenital epithelium [7]. It is characterised by overexpression of proinflammatory Th1 cytokines and a dense T‐cell infiltrate [7], processes that also underpin other autoimmune diseases with which lichen sclerosus may coexist [8]. This tendency towards autoimmunity was demonstrated by concomitant irAEs in all our patients. Previous case reports of adverse effects with ICIs have not described the development of lichen sclerosus in conjunction with other irAEs.

Immune checkpoint blockade disrupts normal immune tolerance by blocking PD‐1, CTLA‐4 and LAG‐3 pathways that usually suppress autoreactive T‐cell activity [1]. Through enhancing immune surveillance and T‐cell activation, ICIs reduce self‐tolerance and therefore may precipitate autoimmune diseases or exacerbate pre‐existing subclinical autoimmune conditions.

A notable feature of our cohort was the persistence of ICI‐associated vulval lichen sclerosus despite standard topical corticosteroid therapy. Most of our patients had difficult‐to‐control disease, even though they had appropriate treatment. In comparison with the sample of patients with idiopathic vulval lichen sclerosus described by Wu et al. [9], whose VQLI scores improved to a median of 5/45 with treatment, the high VQLI burden in our ICI‐associated cohort (10/45 despite treatment) suggests a potentially more refractory disease phenotype. The suggestion that ICI‐associated disease may be more refractory possibly reflects enhanced or sustained immune activation triggered by ICIs. This contrasts with a previously described case, where rapid resolution was observed with ultrapotent topical corticosteroid treatment [2].

The latency between ICI initiation and lichen sclerosus diagnosis was frequently prolonged. The latency periods of irAEs are not well documented but appear to be highly variable [1, 10]. The prolonged latency between ICI initiation and vulval lichen sclerosus diagnosis may be partly explained by under‐recognition. The delayed onset may also reflect the chronic, slowly progressive nature of lichen sclerosus. Furthermore, vulval examination is not routine during oncology follow‐up, and patients may be reluctant to report symptoms due to embarrassment. Increased awareness among oncologists and dermatologists is essential to improve detection and management of this irAE.

Another important observation was that the biopsies from post‐ICI cases showed similar classical features to idiopathic vulval lichen sclerosus. This suggests that while ICIs may act as a trigger for disease onset, the downstream pathological processes resemble those of idiopathic disease. For pathologists, this is diagnostically reassuring as ICI‐associated disease does not appear to demonstrate atypical features but instead the same histopathological hallmarks as idiopathic cases. Our findings appear consistent with the histopathology findings of a single case of post‐ICI vulval lichen sclerosus reported by Schaberg et al., in which a vulval punch biopsy showed epidermal atrophy, hyalinised dermal collagen and a dense lymphocytic infiltrate [11], classical features diagnostic of lichen sclerosus. Taken together, these data indicate that histopathology may support the diagnosis of vulval lichen sclerosus when the disease is suspected clinically, but the distinction between idiopathic and ICI‐associated disease rests on correlation with treatment history and timing of symptom onset. However, since the number of biopsy‐confirmed cases in our cohort was small (6/11), subtle histological differences between ICI‐associated and idiopathic lichen sclerosus cannot be excluded.

The study is not without limitations. Firstly, idiopathic lichen sclerosus occurs more commonly in post‐menopausal women, who constituted the vast majority of our sample. Therefore, establishing specific causality with ICI exposure is challenging. Secondly, data about vulval symptoms or lichen sclerosus are not systematically collected on the Melanoma Research Database. Cases may therefore be missed because lichen sclerosus is not a defined irAE and thus clinicians may not routinely screen for it.

To the best of our knowledge, this is the largest reported series of vulval lichen sclerosus associated with ICI therapy. Our findings support an association between ICIs and lichen sclerosus. The growing use of immunotherapies for cancer and paucity of published information about lichen sclerosus that may develop in some treatment recipients heightens the need to expand our knowledge about this side effect. Prospective evaluation, incorporating routine enquiry about vulval symptoms and use of validated tools such as the VQLI, may help to better define the incidence, natural history and outcomes of ICI‐associated lichen sclerosus. Awareness of the potential occurrence of ICI‐associated lichen sclerosus is important to facilitate specific screening, support early diagnosis, initiate necessary treatment to relieve symptoms and mitigate the low risk of malignant transformation.

## Ethics Statement

The study was approved by the Human Research Ethics Committee of the Sydney Local Health District Ethics Review Committee (X15‐0311 and 2019/ETH06854).

## Conflicts of Interest

A.M.M. has served on advisory boards for B.M.S., M.S.D., Novartis, Pierre‐Fabre, Roche and QBiotics. G.V.L. is a consultant advisor for Agenus, AstraZeneca, Bayer, BioNTech, Boehringer Ingelheim, Bristol Myers Squibb, Byondis B.V., Evaxion, Fortiva Biologics, GI Innovation, Hexal AG (Sandoz Company), Highlight Therapeutics S.L., Immunocore, Innovent Biologics USA, IOBiotech, Iovance Biotherapeutics, MSD, Novartis, Pierre Fabre, Regeneron, Scancell, SkylineDX B.V. and Strand.

## Supporting information


**Data S1:** ajd70058‐sup‐0001‐Supinfo.docx.

## Data Availability

The data that support the findings of this study are available from the corresponding author upon reasonable request.
