# Supplementary material for: Vulval Lichen Sclerosus Associated With Immune Checkpoint Inhibitor Therapy: A Case Series of 11 Patients
Source: Australas J Dermatol. 2026 Feb 2;67(3):176–80. doi: 10.1111/ajd.70058 (PMC13176800; doi:10.1111/ajd.70058)
Supplement: Supplementary file 1 — Data S1: ajd70058‐sup‐0001‐Supinfo.docx. [file AJD-67-176-s001.docx]

**Supporting Information**

**Supporting Information 1. Questionnaire completed by patients about their history**

| Which of the following symptoms did you experience when you were diagnosed with lichen sclerosus (you may select multiple)? | - Itch - Difficulty with urination - Bruising more easily than before - Fragile skin - Burning sensation - Soreness - Other - None of the above |
| --- | --- |
| If other, please specify: |  |
| Did you have these symptoms before starting immunotherapy (you may select multiple)? | - Itch - Difficulty with urination - Bruising more easily than before - Fragile skin - Burning sensation - Soreness - Other - None of the above |
| If other, please specify: |  |
| If you had a history of vulval lichen sclerosus prior to commencing immune checkpoint inhibitor therapy, during the time you were receiving immune checkpoint therapy for your melanoma, did you experience any change in your lichen sclerosus symptoms? | - Worsening - Improvement - No change |
| How long was it between the onset of any of these symptoms and the time you sought medical attention? |  |
| How long was it between the onset of any of these symptoms and the time you received the diagnosis of lichen sclerosus? |  |
| Who made the diagnosis of lichen sclerosus? | - GP - Dermatologist - Oncologist - Gynaecologist - Other |
| If other, please specify: |  |
| Which of the following methods were used to make the diagnosis of lichen sclerosus (you may select multiple)? | - Clinical examination - Photograph of your skin - Skin biopsy - Other |
| If other, please specify: |  |
| If you had a skin biopsy to diagnose your lichen sclerosus, when and by whom was it performed? |  |
| If applicable, at what age (in years) did you experience menopause? |  |
| Do you have a history of autoimmune disease? | - Yes - No |
| If yes, please specify what autoimmune conditions you suffer from: |  |
| Do you have a family history of autoimmune disease (in blood relatives)? | - Yes - No |
| If yes, which autoimmune conditions affect or affected members of your family (blood relatives)? |  |
| What treatment did you receive for lichen sclerosus? |  |
| How have you responded to the treatment for your lichen sclerosus? | - My symptoms have completely resolved - My symptoms have improved with treatment, but I still experience symptoms - My symptoms are the same despite treatment - My symptoms have worsened despite treatment - Not applicable – I have never been on treatment for lichen sclerosus |
| **Vulval Quality of Life Index (VQLI) Questionnaire** |  |
| 1. Over the past month how itchy and/or painful and/or stinging and/or burning has your vulvar skin felt? | - Very much - A lot - A little - Not at all |
| 1. Over the past month, how often have you experienced any of the following: pain when urinating, heat intolerance, vaginal discharge, wetness? | - Very much - A lot - A little - Not at all |
| 1. Over the last month how embarrassed or self-conscious have you been because of your vulvar skin symptoms? | - Very much - A lot - A little - Not at all |
| 1. Over the past month how much has your vulvar skin impacted your body image or sense of self? (For instance, sense of femininity, feeling isolated, feeling different)? | - Very much - A lot - A little - Not at all |
| 1. Over the last month how distressed or anxious have you felt because of your vulvar skin problem? | - Very much - A lot - A little - Not at all |
| 1. Over the last month how much has your vulvar skin problem influenced your choice of clothing (For instance underwear, jeans, gym clothes)? | - Very much - A lot - A little - Not at all |
| 1. Over the last month how much has your vulvar skin problem disturbed your sleep? | - Very much - A lot - A little - Not at all |
| 1. Over the last month how much has your vulvar skin problem made it difficult for you to go shopping, look after yourself or your family, home and garden? | - Very much - A lot - A little - Not at all |
| 1. Over the last month how much has your vulvar skin problem made it difficult for you to attend social or leisure engagements? (For instance, going out for dinner or bars, dating, sport, exercise class, gym) | - Very much - A lot - A little - Not at all |
| 1. Over the last month how much has your vulvar skin problem interfered with your ability to concentrate on work or study? | - Very much - A lot - A little - Not at all |
| 1. Over the last month how much has your vulvar skin problem created problems with a partner or precluded you from pursuing a romantic relationship? (For instance, maintaining a relationship or finding a partner) | - Very much - A lot - A little - Not at all |
| 1. Over the last month how much has your vulvar skin problem interfered with your sex life? (For instance. decreased libido, decreased frequency of sex, pain with sex and/or enjoyment of sex) | - Very much - A lot - A little - Not at all |
| 1. Over the last month how often have you felt distressed or worried about sex because of your vulvar skin? | - Very much - A lot - A little - Not at all |
| 1. How often in the last month have you been worried about long-term health implications of your vulvar skin condition? (For instance, concern about developing cancer or difficulties with fertility) | - Very much - A lot - A little - Not at all |
| 1. Over the past month, how much of a problem has the treatment of your vulvar symptoms been (For instance messy, time consuming, expensive, inconvenient)? | - Very much - A lot - A little - Not at all |

**Supporting Information 2. Histopathological features assessed**

| **Histopathological feature** | **Score** |
| --- | --- |
| Lichenoid inflammation | Absent  Present: mild, moderate, marked  NA |
| Vacuolar interface change | Absent  Present: mild, moderate, marked  NA |
| Hyaline fibrosis | Absent  Present: mild, moderate, marked  NA |
| Dermal oedema | Absent  Present: mild, moderate, marked  NA |
| Band-like inflammation deep to sclerosus | Absent  Present: mild, moderate, marked  NA |
| Acanthosis | Absent  Present: mild, moderate, marked  NA |
| Epidermal thinning | Absent  Present: mild, moderate, marked  NA |
| Subepidermal clefting | Absent  Present: mild, moderate, marked  NA |
| Thickness of fibrosis (mm) |  |
| Dermal inflammation | Absent  Present: mild, moderate, marked  NA |
| Perivascular inflammation | Absent  Present: mild, moderate, marked  NA |
| Cell types present (lymphocytes, macrophages, histiocytes, plasma cells, eosinophils) |  |
| Melanophages | Absent  Present |
| Ectatic vessels in dermis | Absent  Present |
| Epidermal atypia | Absent  Present |
| Civatte bodies | Absent  Present |
| Hypergranulosis | Absent  Present: mild, moderate, marked  NA |
| Hyperkeratosis | Absent  Present: mild, moderate, marked  NA |
| Parakeratosis | Absent  Present: mild, moderate, marked  NA |
| Spongiosis | Absent  Present: mild, moderate, marked  NA |
| Intraepidermal inflammation | Absent  Present: mild, moderate, marked  NA |
| Other comments |  |

**Supporting Information 3.**

1,056 female patients received immune checkpoint therapy from 2009 to 2024 in the Melanoma Research Database N=5

Patients identified by clinicians at sites included in Melanoma Research Database N=6

11 patients with vulval lichen sclerosus identified

2 patients with vulval lichen sclerosus had pre-exisiting disease

9 patients developed vulval lichen sclerosus after initiation of immune checkpoint inhibitor therapy

2/2 responded to the questionnaire

9/9 completed VQLI*

8/9 responded to the questionnaire

4/9 had histopathology diagnosis of vulval lichen sclerosus

2/2 completed VQLI

2/2 had histopathological diagnosis of vulval lichen sclerosus

**Figure. Flowchart of case identification for vulval lichen sclerosus in female melanoma patients treated with immune checkpoint inhibitors.**

*For the one patient that did not complete the questionnaire, the VQLI was obtained from the medical record from the most recent clinical follow-up.

VQLI=Vulval Quality of Life Index

**Supporting Information 4.**

**
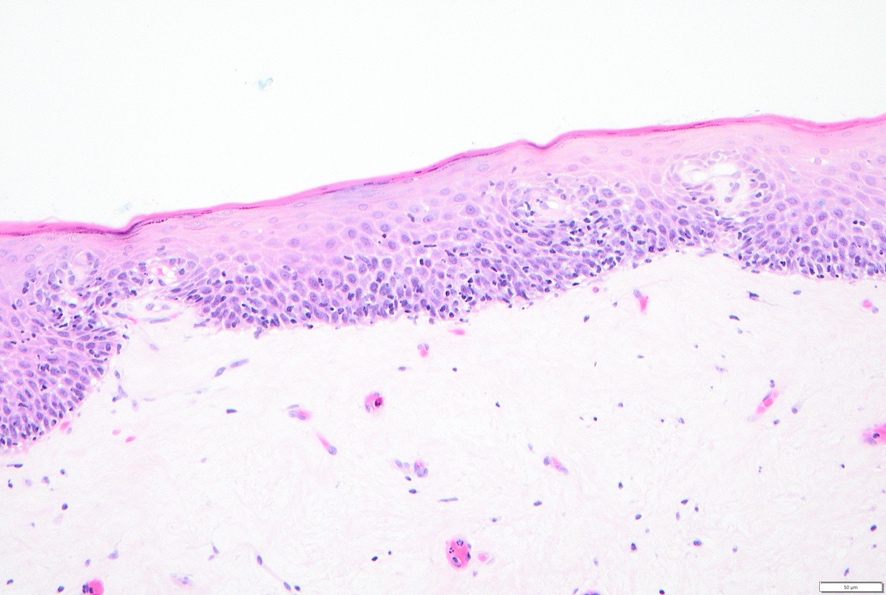
**

**A**

**
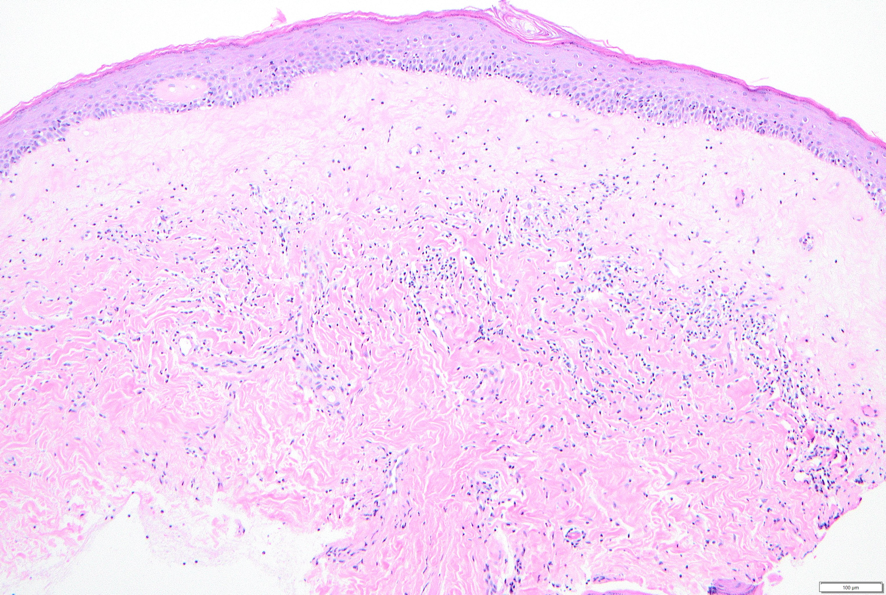
**

**B**

**
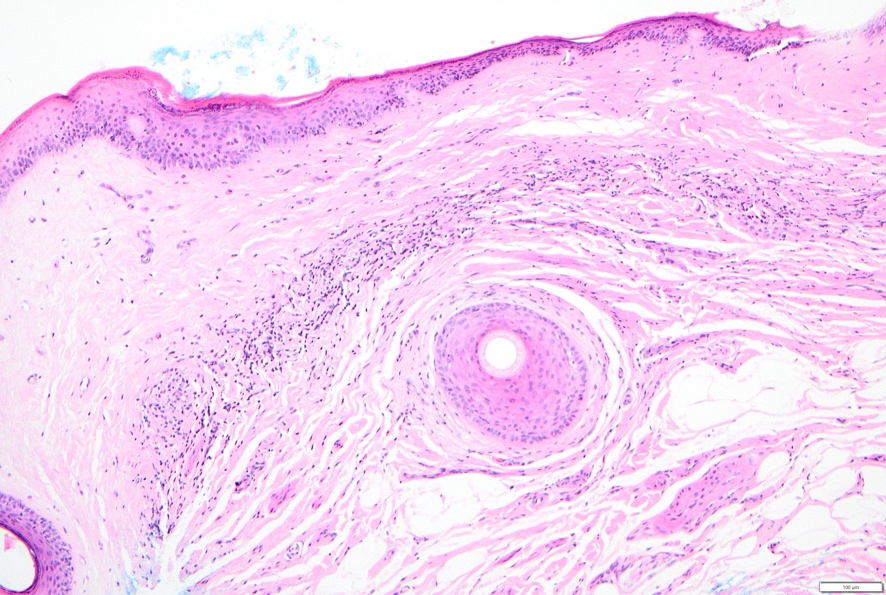
**

**C**

**Figure. Vulval biopsies from post immune checkpoint inhibitor associated vulval lichen sclerosus cases (haematoxylin and eosin stain). (A) Mild to moderate lichenoid inflammation, intraepidermal inflammation and dermal oedema. (B) Dermal fibrosis and oedema. (C) Dermal hyaline fibrosis with underlying band-like inflammation.**
